# Prenatal Diagnosis of a Congenital Postaxial Longitudinal Limb Defect: A Case Report

**DOI:** 10.1155/2010/825639

**Published:** 2010-05-30

**Authors:** Joana Pauleta, Maria Antonieta Melo, Luís Mendes Graça

**Affiliations:** Ultrasound Unit, Department of Obstetrics, Gynecology and Reproductive Medicine, Santa Maria University Hospital, 1649-035 Lisbon, Portugal

## Abstract

*Introduction*. Although congenital longitudinal fibular deficiency is one of the most common long bone deficiencies, there are few published cases of its prenatal diagnosis. *Case report*. A right longitudinal deficiency of the fibula associated with tibial shortening, foot equinovalgus, and absence of the fourth and fifth foot rays diagnosed at 22 weeks gestation is described. Sequential ultrasonographic surveillance was performed without obstetric complications. The anomaly was confirmed after birth, and conservative orthopaedic management was decided. *Conclusion*. Though rarely seen, postaxial longitudinal limb defect may be detected by ultrasound. The correct approach can only be decided after birth, when the functional impact of the anomaly can be fully evaluated.

## 1. Introduction

Postaxial longitudinal defect is one of the most common congenital limb reduction defects. This entity includes a large spectrum of abnormalities that may range from severe hypoplasia to complete absence of the fibula and the 5th rays. It is a rare disorder, with an estimated prevalence of 5.7 to 20 cases per 1 million births [1]. Up to now, only few cases of prenatal diagnosis of isolated longitudinal deficiency of the fibula were reported [1–4].

## 2. Case Report

**Figure 1:**
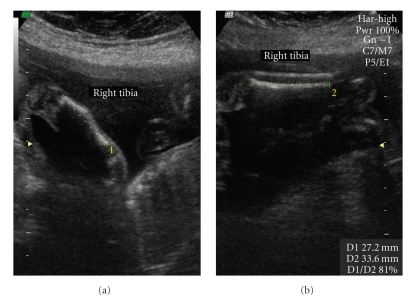
Ultrasound images showing absence of right fibula, bowing of right tibia (a), and tibial discrepancy (right tibia: 27.2 mm and left tibia: 33.6 mm) at 23 weeks gestation (b).

**Figure 2:**
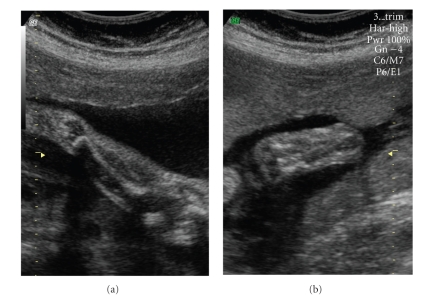
Ultrasound images showing absence of right fibula (a) and absence of the fourth and fifth right foot rays and digits (b) at 31 weeks gestation.

A 38-year-old healthy primigravida, with no familiar history of limb defects or exposure to teratogenic drugs, was referred to our ultrasound unit at 22 weeks gestation after the absence of the right fibula was diagnosed during the second trimester ultrasound examination. This diagnosis was confirmed in our department. Further assessment of the ipsilateral lower limb detected a discrete femur shortening (35 mm versus 37 mm of left femur, discrepancy of 5.4%), anteromedial bowing and tibial shortening (27.2 mm versus 33.6 mm of left tibia, discrepancy of 19%), see Figure 1, foot equinovalgus, and absence of the fourth and fifth foot rays and digits. All other long bones (humeri, ulnae, and radii) were symmetric and appropriated in length and configuration for gestational age, as were the hands. No other anomalies were detected, namely, craniosynostosis, omphalocele, renal displasia, neural tube defects, thoracoabdominal schisis, or facial dysmorphies. Amniocentesis revealed a normal female karyotype (46, XX). Fetal echocardiography was normal. Follow-up ultrasound examinations were carried out periodically until birth (Figure 2). Tibial discrepancy increased slightly with a difference of 13 mm (23.6%) at 34 weeks gestation.

**Figure 3:**
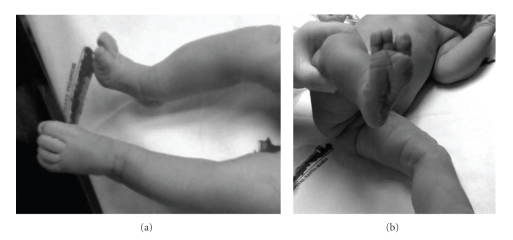
Neonatal pediatric examination at the 1st postnatal day: right limb shortening and bowing (a) and right foot with three digits (b).

**Figure 4:**
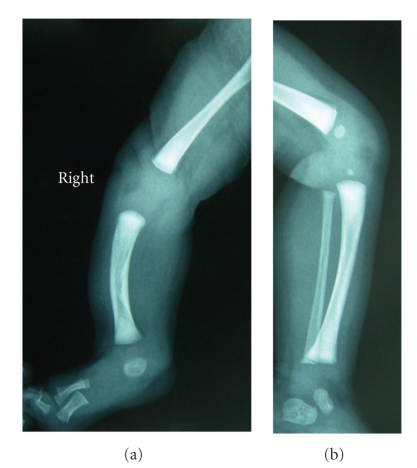
Radiograph showing absence of right fibula and shortening and bowing of right tibia at the 7th postnatal day.

No other antenatal problems occurred, and at 41 weeks gestation a cesarean section was performed due to cephalopelvic disproportion. A 3430 g female newborn was delivered with Apgar scores of 9 and 10 at 1 and 5 minutes, respectively. Neonatal examination (Figure 3) and X-Ray (Figure 4) confirmed the anomalies. Right tibia appeared shortened and bowed anteriomedially, and an ipsilateral equinovalgus foot was present with the absence of the fourth and fifth rays. In spite of limb-length shortening and alterations in limb alignment and stability, normal active mobility of both limbs was observed. No other congenital abnormalities were detected. The newborn was discharged at the fourth postpartum day. The baby was followed up by orthopaedic pediatrics, and there were no perinatal complications. At ten months the infant could sit but not crawl, and she started to walk at the fifteenth month, though she has a normal intellectual development for her age. Orthopaedic surveillance will be maintained and treatment will only be applied when and if needed.

## 3. Discussion

Some authors [5, 6] consider postaxial longitudinal defect the most common lower limb congenital deficiency although there are published data [7, 8] that refer to terminal transverse defects as being the most common one. The inexistence of a consensual data may reflect the rarity of limb malformations, differences between populations, and the lack of uniform classification system of limb reduction defects (LRDs). There are many classification systems for LRD published in the literature, such as the Frantz and O' Rahilly classification [9] and the Achterman and Kalamchi classification [10], which is based on clinical and radiographic findings. European Surveillance of Congenital Anomalies (EUROCAT) proposed a more consistent classification, that was reviewed in 2004. Using the EUROCAT classification, our case report can be classified as a postaxial (fibula) longitudinal defect. This entity includes a large spectrum of abnormalities that may range from severe hypoplasia to the complete absence of fibula and the 5th rays. No sex differences were reported, although some studies referred that males are affected twice as often as females. Moreover, unilateral involvement occurred in two thirds of cases with the right side being more frequently affected [5, 6, 10, 11].

There is a consensus about the critical embrionary period of limb development, which is between 4 and 8 weeks gestation, but there is no precise understanding of the etiology of this unusual disorder. The majority of the cases appeared sporadically or as isolated events although it may be part of a malformative syndrome related with genetic conditions, teratogenic insults, or vascular disruption [11, 12]. A familiar history of skeletal anomalies may be found by Calzolari et al. in 7.2% of all limb reduction defects (LRDs) [7].

Femur-fibula-ulna complex and proximal femoral focal deficiency should be ruled out during the ultrasound examination, as both can show fibular deficiency. Thus, as soon as some fibular deficiency is found, meticulous search for additional long bones and hands abnormalities should be performed. Since there are only few cases associated with other nonskeletal malformations (0.8%) [11], such as cranial, facial, gastrointestinal, urogenital, cardiac, lung, diaphragmatic, and neural tube abnormalities, attention should also be paid to their ultrasound screening. In our case, these malformations were excluded.

Fibular deficiency is generally accompanied by a constellation of other ipsilateral lower limb malformations, with a wide variety of manifestations, such as femoral hypoplasia, tibial a/hypoplasia or anterior bowing, knee and ankle deformities and abnormal positions (equinovalgus), and, finally, toe deficiency, absence of one or more lateral rays [10]. The degree of tibial shortening increases as the fibular deficiency becomes more marked, while femoral shortening did not correlate with the fibular deficiency [10]. The correct evaluation of the severity of foot abnormalities, which appeared to be correlated with the severity of fibular deficiency, is important for assessment of orthopaedic impact.

The diagnosis of lower limb malformations used to be performed, almost always, after birth. Nowadays, it can be identified if a complete, detailed, and skilled prenatal ultrasound examination of lower limbs is systematically done. This highlights ultrasound accuracy for the diagnosis of the postaxial longitudinal defects. We should point out that it may be difficult to determine whether the bone of distal lower extremity is the fibula or the tibia; tibia articulates with the femur.

Bone echodensities seen before pregnancy correlate well with neonatal X-Ray densities, allowing a correct classification of this entity, undoubtedly necessary to orthopaedic assessment. That was clearly shown by the excellent agreement observed in our case between prenatal ultrasound and neonatal radiographic results.

This condition, although not fatal, has a poor prognosis that depends on the degree of limb deformity and on the chosen treatment. Treatment options should be individualized (tibial lengthening, amputation, epiphysiodesis, or prosthetic rehabilitation), and management should be based on careful interpretation of functional, psychological, and cosmetic needs.

To conclude, accurate prenatal ultrasound diagnosis of postaxial longitudinal defect may be performed. The correct approach and advice would be done only after birth, when the impact of the anomaly can be completely evaluated. A multidisciplinary approach including obstetricians, geneticists, neonatologists, and pediatric orthopaedists should always discuss the implications of this situation with the parents wand counsel thoroughly concerning the management, treatment options, and prognosis.
